# Soil protist function varies with elevation in the Swiss Alps

**DOI:** 10.1111/1462-2920.15686

**Published:** 2021-08-10

**Authors:** Florent Mazel, Lucie Malard, Hélène Niculita‐Hirzel, Erika Yashiro, Heidi K. Mod, Edward A. D. Mitchell, David Singer, Aline Buri, Eric Pinto, Nicolas Guex, Enrique Lara, Antoine Guisan

**Affiliations:** ^1^ Department of Ecology and Evolution University of Lausanne Lausanne 1015 Switzerland; ^2^ Department of Occupational Health and Environment Center for Primary Care and Public Health (Unisanté), University of Lausanne Epalinges CH‐1066 Switzerland; ^3^ Center for Microbial Communities, Section of Biotechnology, Aalborg University Aalborg Denmark; ^4^ Department of Geosciences and Geography University of Helsinki Helsinki Finland; ^5^ Laboratory of Soil Biodiversity Institute of Biology, University of Neuchâtel, Rue Emile‐Argand 11 Neuchâtel CH‐2000 Switzerland; ^6^ Jardin Botanique de Neuchâtel, Chemin du Perthuis‐du‐Sault 58 Neuchâtel CH‐2000 Switzerland; ^7^ UMR CNRS 6112 LPG‐BIAF, Université d'Angers Angers Cedex 1 France; ^8^ Institute of Earth Surface Dynamics, University of Lausanne Lausanne 1015 Switzerland; ^9^ Terrabiom Association, Dörflistrasse 32, Oberrieden Zürich 8942 Switzerland; ^10^ Bioinformatics Competence Center, University of Lausanne Lausanne Switzerland; ^11^ Real Jardín Botánico, CSIC, Plaza de Murillo 2 Madrid 28014 Spain

## Abstract

Protists are abundant and play key trophic functions in soil. Documenting how their trophic contributions vary across large environmental gradients is essential to understand and predict how biogeochemical cycles will be impacted by global changes. Here, using amplicon sequencing of environmental DNA in open habitat soil from 161 locations spanning 2600 m of elevation in the Swiss Alps (from 400 to 3000 m), we found that, over the whole study area, soils are dominated by consumers, followed by parasites and phototrophs. In contrast, the proportion of these groups in local communities shows large variations in relation to elevation. While there is, on average, three times more consumers than parasites at low elevation (400–1000 m), this ratio increases to 12 at high elevation (2000–3000 m). This suggests that the decrease in protist host biomass and diversity toward mountains tops impact protist functional composition. Furthermore, the taxonomic composition of protists that infect animals was related to elevation while that of protists that infect plants or of protist consumers was related to soil pH. This study provides a first step to document and understand how soil protist functions vary along the elevational gradient.

## Background

Soil microorganisms are extremely diverse and play key roles in soil nutrient cycling and plant growth (Bardgett and Van Der Putten, 2014). Among these, protists (nonfungal microbial eukaryotes) are important contributors to nutrient cycling and soil respiration (Adl and Gupta, 2006; Margerison *et al*., 2020). Phototrophic protists fix atmospheric carbon and thus participate in the carbon cycle (Geisen *et al*., 2020). Predatory protists [‘consumers’, i.e. individuals that eat other live individuals (Adl *et al*., 2019)] modulate populations of bacteria, archaea, fungi, algae and micro‐metazoan and release nutrients from their prey, controlling soil fertility and plant growth (Bonkowski, 2004; Koller *et al*., 2013; Gao *et al*., 2019). Finally, parasitic protists control plant and animal host population, thereby having an impact on biodiversity and even human economy. For example, the oomycete *Phytophthora infestans*, a well‐known potato pathogen, caused the Irish famine in the 19th century that forced millions of peoples to migrate from Europe to America (Large, 1940). Despite their well‐documented functions in the soil ecosystem (Geisen, 2018), we still have a limited understanding of the spatial and temporal variability of the functional composition of protist soil communities (Geisen *et al*., 2017). In particular, there is a need to better document how functional group abundances vary across main climatic and edaphic gradients. Gathering this information is essential for making predictions about how soil organisms and associated biogeochemical cycles will be impacted by changing environmental conditions (Fierer, 2017).

Recent studies have shown that soil protist communities are mainly composed of consumers, parasites and – to a lesser extent – phototrophs and that their relative abundances vary widely across habitats and across the globe (Oliverio *et al*., 2020; Seppey *et al*., 2020). For example, Mahé *et al*. (2017) found that tropical forest soils host a high abundance of parasitic lineages while Oliverio *et al*. (2020) found that temperate soil habitats are enriched in consumers. Protist functional group abundance varies also across habitats at local scales: for example, between forest and adjacent agricultural field (Schulz *et al*., 2019) or grassland (Fiore‐Donno *et al*., 2020). Within a given habitat type, functional group abundance also varies in relation to soil properties [e.g. soil amendment in agricultural fields, (Xiong *et al*., 2018)] and between seasons (Fiore‐Donno *et al*., 2019). However, it is still unclear how protist functional group composition varies across the elevational gradient. Mountains slopes represent one of the steepest environmental gradients found on Earth and have fascinated biogeographers and ecologists for centuries (von Humbolt and Bonpland, 1805; Hoorn *et al*., 2018). Mountains are biodiversity hotspots and represent ideal study systems to understand the processes shaping biological diversity (Rahbek *et al*., 2019). While the taxonomic and functional compositions of many groups of organisms have been documented on mountain slopes (Rahbek *et al*., 2019), very little is known for soil protists. A recent study has shown that soil protists richness was shown to vary broadly across elevation and to be correlated to both climatic and edaphic factors (Seppey *et al*., 2020) but no study, to the best of our knowledge, has documented how the composition of soil protist functional groups varies with elevation.

One way to fill this gap is to use the knowledge accumulated on the function of each taxon and to pair it with the power of next‐generation sequencing to attribute the function to identified taxa (Adl *et al*., 2019). Such an approach was successfully applied for protists in several recent studies (Schulz *et al*., 2019; Oliverio *et al*., 2020), and can therefore also be used to document how functional group abundance and composition vary with elevation. Theory predicts that parasite abundance should decrease toward mountain tops as their specific hosts' (or vectors') biomass and/or abundance decreases (van Riper *et al*., 1986; Zamora‐Vilchis *et al*., 2012). Moreover, parasite taxonomic community composition should be correlated to protist hosts' community composition while phototroph or consumer taxonomic community composition should be more correlated to the abiotic environment (Ohlmann *et al*., 2018).

Here, using the taxonomical information provided by the metabarcode sequencing of soil samples from 161 open habitats spanning 2600 m of elevation (from 400 m to ca. 3000 m), we ask four questions: (i) what is the relative proportion of DNA sequences of three functional groups (parasites, consumers and phototrophs) in open habitats? (ii) How do these proportions vary across the elevational gradient? (iii) Which environmental factor(s), including plant community composition, best explains changes in functional group composition along the elevational gradient? (iv) How does protist community taxonomic composition vary within functional groups along the elevational gradient?

## Methods

### Soil sample collection

The study region (700 km^2^) is located in the western Swiss Alps. Open habitats soils sampling was performed in summer 2013, as described in detail in Yashiro *et al*. (2016). Briefly, from each plot, five soil cores were taken from the four corners and the centre of a 4 m^2^ plot, pooled in a sterile bag and kept at 4 °C until DNA extraction (within 36 h) and soil analyses were done.

### Amplicon 18s rRNA gene sequencing

DNA extractions, PCR and sequencing procedures were described in Seppey *et al*. (2020). Briefly, DNA was extracted from the soil samples using the MoBio PowerSoil DNA extraction kit following the manufacturer's instructions. The V4 region of the 18S rRNA gene was then amplified in triplicates using the general eukaryotic primers TAReuk454FWD1 and TAReukREV3 (Stoeck *et al*., 2010). PCR conditions and quantification of the amplicon were described in detail in Seppey *et al*. (2020). Sequencing was performed with an Illumina® MiSeq (TruSeq Nano PCR‐free Library Preparation Kit and the paired‐end 2 × 300 bp) at the University of Geneva (Molecular Systematics & Environmental Genomics Laboratory). Sequences are available on European Nucleotide Archive via the project number PRJEB30010 (ERP112373).

### Sampling sites environmental characterization

Climatic and edaphic conditions were obtained for each plot. Below, we briefly describe how they were measured or derived from climatic models but more details can be found in published studies that have already used the same data (Yashiro *et al*., 2016, 2018; Buri *et al*., 2020; Seppey *et al*., 2020). As we were mainly interested in documenting variation of protist community composition across the elevational gradient, we intentionally included elevation as a covariate, along with edaphic and climatic factors in our models. However, the biological variation observed along the elevational gradients results from climatic and edaphic parameters co‐varying with elevation. The climatic and edaphic factors were selected based on two criteria: they must (i) be known *a priori* to have an impact on soil microbial community composition, and (ii) be relatively uncorrelated with each other (Spearman rho <0.7). We initially selected seven edaphic variables following (Seppey *et al*., 2020) and climatic variables (https://www.unil.ch/ecospat/en/home/menuguid/ecospat‐resources/data.html#chclim25) at 25m resolution. We then performed a principal component analysis on all 27 environmental factors to extract the axes of main variation. The first axis relates to elevation and other correlated climatic factors (temperature and precipitation) while the second and third axes represent edaphic factors, in particular total organic carbon (TOC) concentration (Additional File 1, Supp. Fig. 1). For subsequent analysis, we included elevation as it was the factor of interest in this study, mean annual temperature (bio1), TOC, soil pH, bulk soil water content (a drainage indicator) and C/N ratio (a biologically relevant summary of nutrient availability) (Additional File 1, Supp. Fig. 2). Finally, we also included plant community type (phytosociological alliances) and plant richness as they can also influence belowground communities (Delarze *et al*., 1998; Fierer, 2017) (Additional File 2). Plant richness was derived for 145 sites from the existing database (Dubuis *et al*., 2011) where all vascular plant species were exhaustively inventoried in each site.

### Bioinformatic analysis

#### Reproducibility of the pipeline

The whole bioinformatic pipeline described below has been written in R (version 4.0.2) (R Development Core Team, 2015) with the use of the tidyverse (version 1.3) (Wickham *et al*., 2019) and the phyloseq packages (version 1.32) (McMurdie and Holmes, 2013). The associated R code is available on *github*: https://github.com/FloMazel/Protist_Altitude_alps.

#### Sequencing data processing and taxonomic annotation

Raw reads were demultiplexed and barcodes and primers were trimmed using cutadapt version 2.9 (with parameters *e* = 0, no‐indels, *m* = 100) (Martin, 2011). Reads were then quality filtered using the *filterAndTrim* dada2 R function (with parameters maxEE = 6, truncQ = 2, truncLen = 0) (Callahan *et al*., 2016). Amplicon sequences variants (ASVs) were inferred using the *dada* function and reads were merged using the *mergePairs* function (with parameter minOverlap = 8). Chimaeras were removed using the *removeBimeraDenovo* dada2 R function. Triplicates were then pooled according to their respective samples (data are provided in Additional File 3). ASVs were chosen instead of cluster of sequences based on an arbitrary threshold (e.g. 97%) because they: (i) represent finer taxonomic units, (ii) allow comparison between studies and (iii) have become the unit of choice in environmental DNA analysis, including protist sequences (Oliverio *et al*., 2020). We assigned taxonomy for each ASV using the naïve Bayesian RDP classifier, as implemented in dada2 (function *assignTaxonomy*, parameter minBoot set to 50) with the PR^2^ database (version 4.12.0 – file ‘pr2_version_4.12.0_18S_dada2’ provided on the dada2 website, data are provided in Additional File 4 along with bootstrap values for each assignation).

#### ASV filtering and functional annotation

**Fig. 1:**
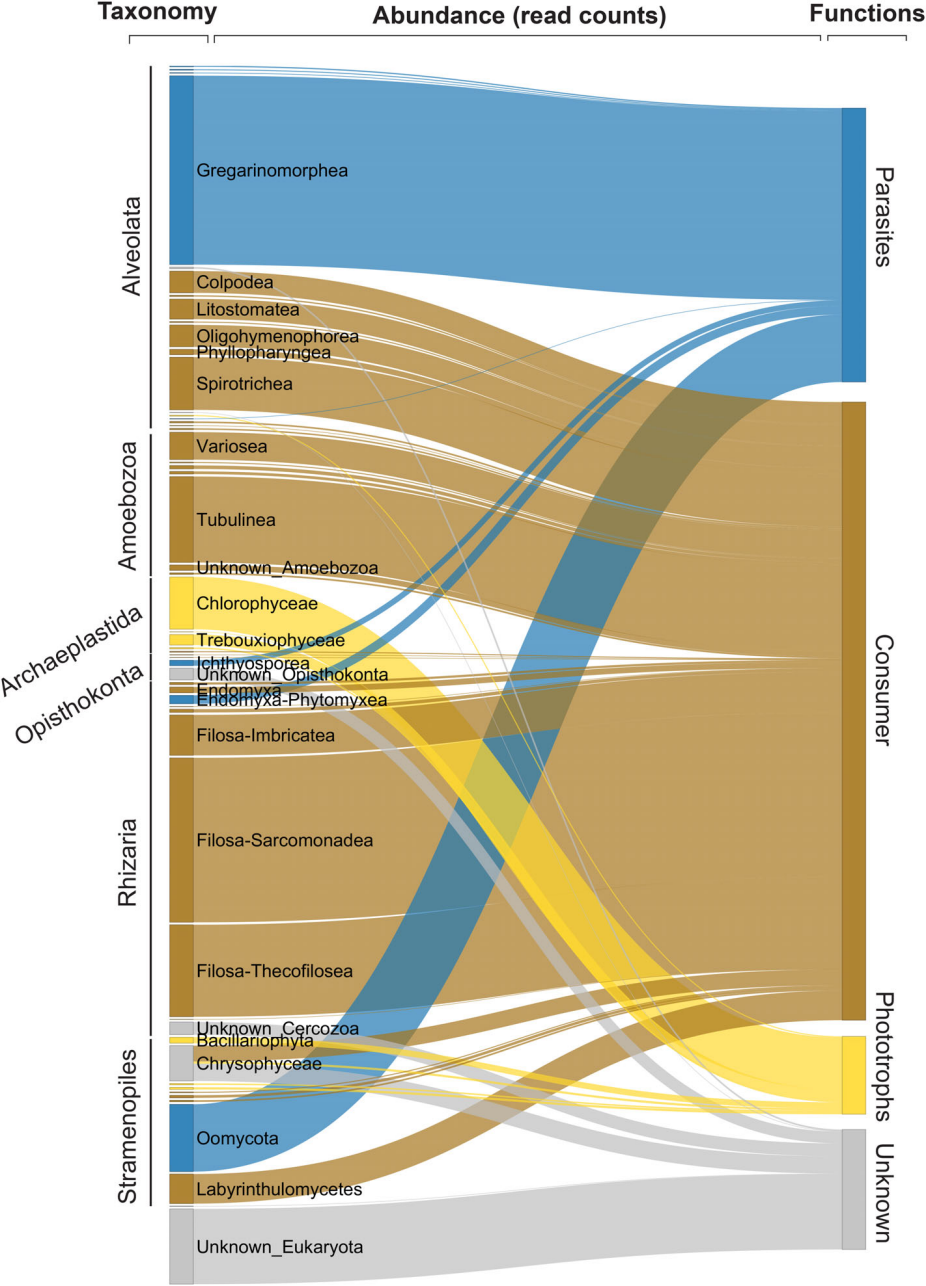
Relationship between protists taxonomy and functions in alpine soils. Various taxonomic groups (rank ‘Class’ nested in rank ‘Supergroup’ in the PR^2^ v 4.12 database, left bars) are connected to their predicted functions (right bars) with links. Link width is proportional to the number of reads assigned to each category in the whole dataset (total reads number = 3 520 752).

Plant, metazoan, fungi and rare protist ASVs (defined as present in only one sample or with fewer than 100 reads overall) were removed. Each remaining ASV was considered as a protist and assigned to one of the three functional groups (Parasites, Consumers or Phototrophs) according to expert knowledge (see Additional File 5 for the assignation rules and Fig. 1 for the result). We acknowledge that these broad functional categories do not distinguish between fine‐scale trophic niche differences. For example, the ‘Consumers’ group encapsulates protists that might feed on very different substrates (fungi, bacteria, other protists) and might either be predators or saprotrophs. Similarly, the ‘Parasites’ group includes protists that can infect a wide range of hosts (e.g. plants or animals). While these broad functional groups represent a gross simplification of protist trophic niches, they can be useful to depict the overall distribution of functional groups across environmental gradients and represent a first necessary step toward the functional characterization of soil protist communities (Geisen *et al*., 2017; Geisen, 2018; Schulz *et al*., 2019; Oliverio *et al*., 2020).

#### Statistical analysis

The relative abundance of the different functional groups along the environmental gradients was visualized by plotting the relative read counts of each group in each sample against environmental factors. However, due to the compositional nature of environmental DNA data, it is impossible to statistically relate these relative abundances to environmental values and ratio of read numbers between groups must be used instead (Gloor *et al*., 2017; Knight *et al*., 2018). We focused on the log‐transformed ratio between consumers and parasites read counts as these two groups are the most abundant ones. To measure the univariate correlation between elevation and this log ratio, we used a Bayesian linear model using the R package MCMCglmm (version 2.29) (Hadfield, 2010) with 100 000 total iterations and 3000 iterations as burn‐in. To further measure the correlation of other environmental factors on this log ratio, we built a linear model using elevation, soil pH, TOC, C/N ratio, mean annual temperature, bulk soil water content (%) and plant alliance and richness as covariates and tested the correlation of each factor using marginal sum of squares and ANOVA with *F*‐values (i.e. testing the correlation of one factor given the correlation of all the other ones, using the *drop1* R function).

Within each functional group, we measured community compositional turnover using a metric of beta‐diversity (Bray–Curtis) on a standardized ASV*sample matrix (each sample counts divided by sample total sum counts). To measure the correlation of the environmental factors on beta‐diversity, we used distance‐based redundancy analysis as implemented in vegan (version 2.5) (Oksanen *et al*., 2016). Each factor effect was tested using randomisation (*n* = 999) and effect sizes were measured using pseudo *F* statistics with marginal sum of squares (function *adonis2* in the R vegan package, argument ‘by’ set to ‘margins’). To be able to compare the outputs of the distance‐based redundancy analysis between functional groups, we selected a common set of samples that had at least 400 read counts for each group. To test the robustness of our result to various sequencing depths between samples and functional groups, we repeated the analysis by rarefying the samples to 350 reads.

## Results

A total of 3 520 752 reads corresponding to 5222 protists ASVs were recovered from 161 random‐stratified open habitats soil samples in the western Swiss Alps. We then assigned each read to a functional group based on taxonomy (Fig. 1). Over the whole study area (700 km^2^), alpine protists communities were dominated by consumers (57% of reads, 64% of ASVs) followed by parasites (25% of reads, 14% of ASVs) and phototrophs (7% of reads, 5% of ASVs), while 11% of reads could not be functionally assigned due to poor taxonomic resolution (Fig. 1).

**Fig. 2:**
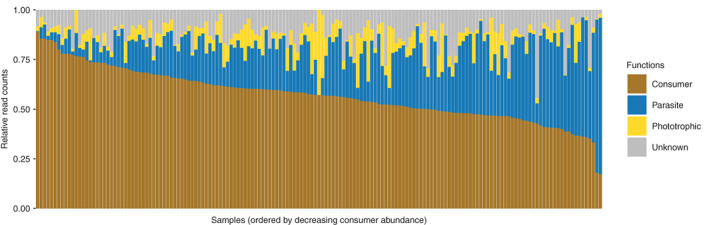
The relative abundance of soil protist functions varies widely among samples. The relative abundance (relative read counts, *y*‐axis) of the different functional groups (colours) is represented across samples (*x*‐axis). Samples (*n* = 145) are ordered by descending relative counts of consumer reads.

**Fig. 3:**
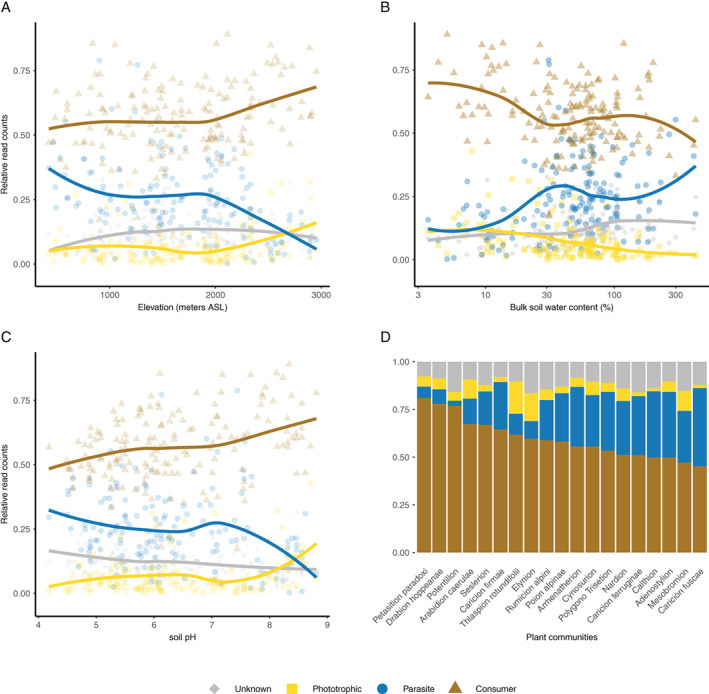
Soil protist functional composition relates to the environment. All panels depict the relationship between functional group relative abundance (relative number of reads per sample) and the environment: either elevation (meters ASL, A), bulk soil water content (%, B), soil pH (C) or plant communities (ordered by descending relative counts of consumer reads, panel D). Trend lines for panels A–C represent a loess fit that is shown for illustrative purpose only.

**Fig. 4:**
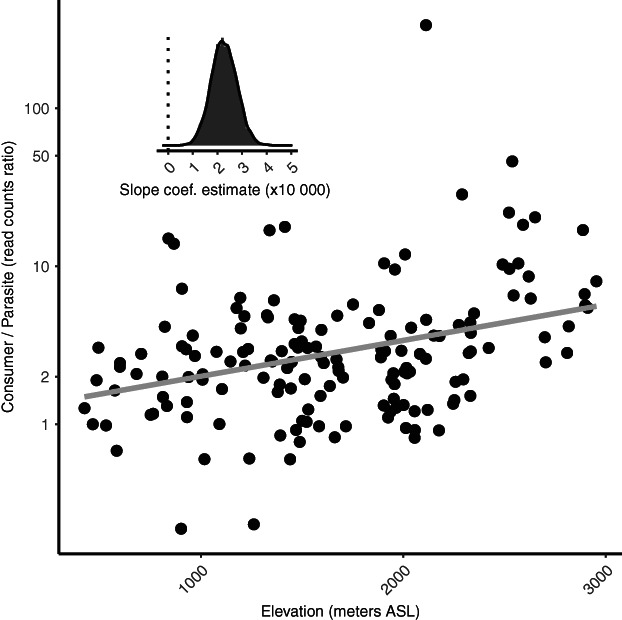
Soil protist consumer and parasite abundance vary widely with elevation. The ratio of read counts between consumer and parasites (Consumer/Parasites, *y*‐axis) is plotted against elevation (*x*‐axis, meters ASL). Inside panel shows the posterior probability of the slope of the relationship depicted in the main panel.

While consumers were over the whole study area the most abundant functional group, their contribution to local (i.e. per sample) read counts varied considerably, between 17% and 89% (Fig. 2). This local variability was correlated to elevation: in general, relative read counts of both consumers and phototrophs increased while those of parasites decreased with elevation (Fig. 3A) and this trend was observed across most consumer and parasites lineages (Supp. Fig. 1). The average ratio of consumer to parasite reads increased from ca. 3 at low elevation (0–1000 m, median ratio = 2) to ca. 12 at high elevation (2000–3000 m, median ratio = 3.6) (Fig. 4, MCMC *p*‐value <0.001). In our data set, elevation was strongly correlated to the climatic gradients (temperature, precipitation) and, to a certain degree, to edaphic (soil pH, bulk soil water content) and biotic (plant communities, plant richness) factors as well (Additional File 1, Supp. Figs 2–3), so that these environmental factors are also related to the reads counts of the different functional groups (Fig. 3B–D, Additional File 1 Supp. Fig. 4). For example, the soil from different plant communities [i.e. plant phytosociological alliances (Delarze *et al*., 1998)] host varying proportions of the three protist groups (Fig. 3D). To measure the correlation between each environmental factor and the ratio of consumer to parasite reads, while taking into account their correlation with the other environmental factors, we proceeded as follows. First, we built a model with all the environmental factors as explanatory variables and the ratio of consumer to parasite reads as the response variable (full model with 145 sites because some of the 161 sites do not have plant data). Second, we dropped each environmental factor independently and one at a time, and measured the fit of the corresponding – reduced – model. Third, we quantified whether the fit of the reduced model was significantly lower than the fit of the full model, in which case the dropped factor was considered significant in the full model. We found that, except for plant alliances, none of the environmental factors had a significant marginal correlation (Additional file 1, Supp. Table 2). In other terms, consumers disproportionally outnumber parasites at high elevations, in alkaline and dry environments and when associated to some plant alliances (e.g. the *Petasition paradoxi* plant alliance).

**Fig. 5:**
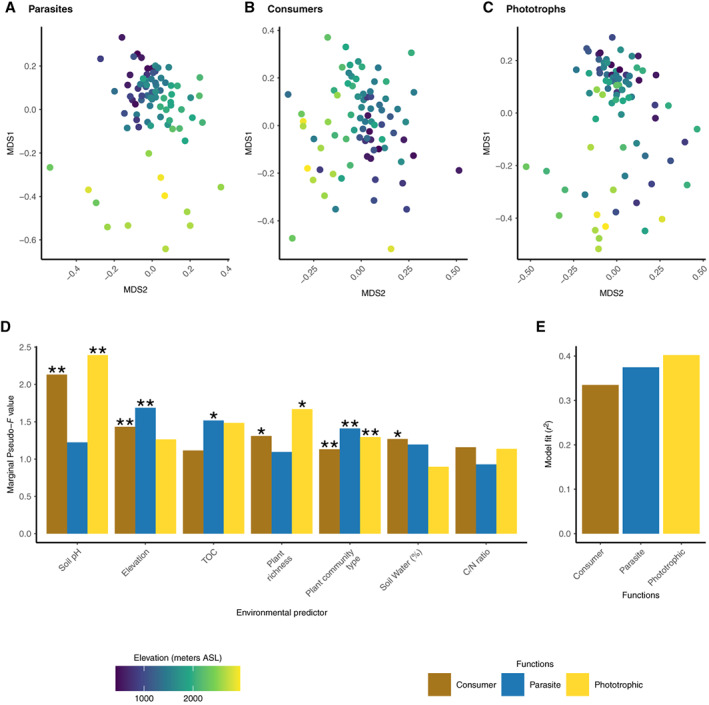
Soil protist community composition within functional groups across the elevation gradient. The figure shows how taxonomic (ASV) compositional turnover varies across the environmental gradient within each of the three functional groups (consumers, parasites, phototrophs). Panels A–C depict NMDS ordination of beta diversity (Bray–Curtis) with samples coloured by elevation for the three functional groups independently (stress values for parasites = 0.18; consumers = 0.22; phototrophs = 0.21). Panels D–E depict the results of distance‐based redundancy analysis based on standardized Bray–Curtis dissimilarities. Panel E presents the general fit of the model (*R* ^2^) and panel D presents individual factor importance, as assessed by pseudo‐*F* values (marginal effect of the factor, i.e. considering all other factors in the model). Statistical significance of each factor is calculated using 999 permutations and indicated with an asterisk (type III sum of squares, *p‐value <0.05, **p‐value <0.01).

**Fig. 6:**
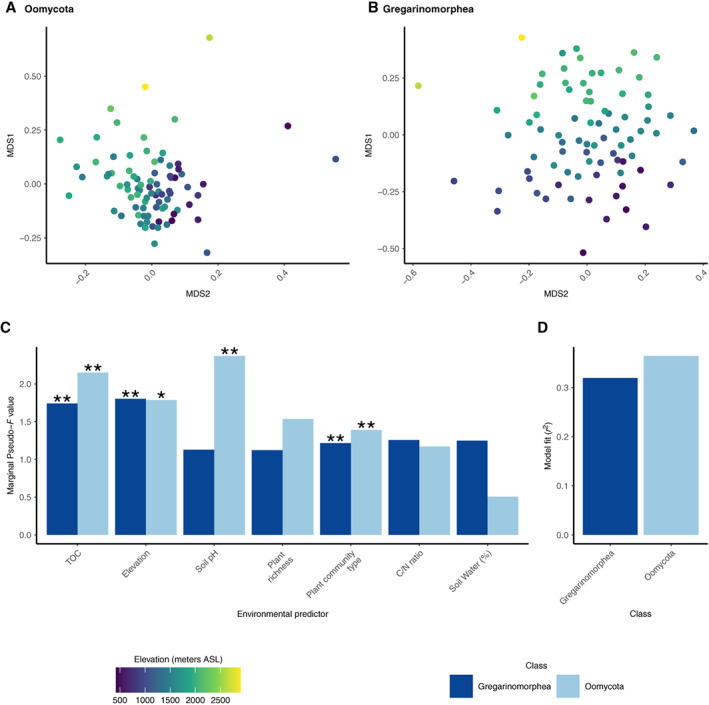
Community composition within parasites across the altitudinal gradient. The figure shows how taxonomic (ASV) compositional turnover varies across the environmental gradient within each of two parasitic clades. Panels A–B depict NMDS ordination of beta diversity (Bray–Curtis) with samples coloured by elevation for the two clades independently (stress values for Gregarinomorphea = 0.26; Oomycota = 0.17). Panels C–D depict the results of distance‐based redundancy analysis based on standardized Bray–Curtis dissimilarities. Panel D presents the general fit of the model (*R* ^2^) and panel C presents individual factor importance, as assessed by marginal pseudo‐*F*‐values (i.e. effect of the factor considering all other factors in the model). Statistical significance of each factor is calculated using 999 permutations and indicated with an asterisk (type III sum of squares, *p‐value <0.05, **p‐value <0.01).

Next, we sought to measure to what extent of taxonomic variability within the functional groups across samples (i.e. taxonomic beta‐diversity) also correlated with elevation and environmental factors. The taxonomic beta‐diversity between samples (as quantified with the Bray–Curtis dissimilarity at the ASV level) within each functional group showed contrasted correlation to elevation (Fig. 5A–C for ordination plot and Fig. 5D for correlation values, Additional file 1 Supp. Fig. 5) and other environmental factors (*R* ^2^ between 0.25 and 0.35, Fig. 5E). For example, soil pH showed higher correlation with consumer (pseudo‐*F* = 2.3, *p*‐value <0.001, Fig. 5D) and phototrophs (pseudo‐*F* = 2.9, *p*‐value <0.001, Fig. 5D) than with parasite taxonomic beta‐diversity (pseudo‐*F* = 1.4, *p*‐value = 0.04, Fig. 5D) and this was similar for the effect of plant richness (Fig. 5). The trend was the opposite for elevation (contrast pseudo‐*F* values in Fig. 5D). Bulk soil water content showed higher correlation with consumer than with parasite or phototrophs taxonomic beta‐diversity (contrast pseudo‐*F* values in Fig. 5D). Within parasites, we distinguished between Gregarinomorphea (a clade of Apicomplexan that usually parasites animals), Oomycota (that are mostly plant parasites) and Endomyxa–Phytomyxea. We found that Gregarinomorphea taxonomic beta‐diversity was mainly correlated to elevation (pseudo‐*F* = 2, *p*‐value <0.001) and TOC (pseudo‐*F* = 1.7, *p*‐value <0.001) while Oomycota taxonomic beta‐diversity was mainly related to soil pH (pseudo‐*F* = 2.45, *p*‐value <0.001) and TOC (pseudo‐*F* = 1.9, *p*‐value <0.001) (Fig. 6 and Additional File 1 Supp. Figs 6–7). For Endomyxa–Phytomyxea, we only recovered a few read per sample (Supp. Fig. 8) that prevented statistical testing but preliminary results suggest that Endomyxa–Phytomyxea taxonomic composition also changes with altitude (Supp. Fig. 8).

## Discussion

Soil protists play unique and multiple functions in soil ecosystems (Geisen, 2018). To understand how these functions vary along major environmental gradients, and especially along elevation, we compared their relative DNA read counts among 161 soil samples representing varying elevations and environmental conditions from open (i.e. non‐forest) mountain habitats (analysis using plant data was done with 145 sites).

We found that consumers dominate soil communities, which is in agreement with previous reports in other temperate soils (Oliverio *et al*., 2020; Seppey *et al*., 2020) but in contrast with tropical soils where parasites seem to predominate (Mahé *et al*., 2017; Schulz *et al*., 2019; Singer *et al*., 2019). The dominance of consumers in mountain open habitat soils suggests that this protist functional group could be key in the cycling and turnover of nutrients in this type of ecosystem (Foissner, 1999; Bonkowski, 2004; Adl and Gupta, 2006; Oliverio *et al*., 2020). While being functionally important and abundant, protist consumers are also extremely diverse (Adl and Gupta, 2006). Indeed, they are known to feed on a wide variety of substrates and to occupy different trophic niches (Geisen, 2018). For example, distinct predators have different preys, including bacteria, fungi, micro‐metazoa and other protists (Glücksman *et al*., 2010; Seppey *et al*., 2017; Geisen, 2018). Given the amplicon sequencing approach taken for this study, it was not possible to finely delineate protist functions, but it could be interesting for future studies to document in more detail the trophic niches of soil protists in order to better sketch out the structure of the regional soil trophic network (Geisen, 2016). Knowledge of the nature of the trophic links between consumer and their substrates will facilitate the measurement of trophic redundancy (i.e. the number of organisms filling the same trophic niche) and vulnerability of the ecosystem to extinction cascades, which may disrupt ecosystem functions (Sanders *et al*., 2018). In addition, documenting local trophic web structure is believed to be key to test theories on how communities assemble and how biotic interactions vary with the environment (Guzman *et al*., 2019). Besides consumers, we also found that parasites account for nearly 25% of the total amplicon reads, mostly represented by Gregarinomorphea and Oomycota, to a lesser extent by Phytomyxea. These reads mostly originate from organisms that infect plant tissues – in particular plant roots for Oomycota ASVs (Thines, 2018) – or soil‐dwelling animals such as insects, other arthropods and earthworms for Gregarinomorphea ASVs [though genera infecting vertebrates are encountered as well in environmental sequences (Singer *et al*., 2020)]. While the broad functional groups used here represent a gross simplification of protist trophic niches, they can be useful to depict the overall distribution of functional groups across environmental gradients and represent a first necessary step toward the functional characterization of soil protist communities (Geisen *et al*., 2017; Geisen, 2018; Schulz *et al*., 2019; Oliverio *et al*., 2020). We also acknowledge that the relative amplicon read counts used in our study is not a direct measure of absolute abundance, biomass nor biological activity of protists in the field but at best describe relative abundances (Taberlet *et al*., 2018). Future work could include direct cell count measurements to corroborate our findings about the dominance of consumers in open habitat soils (Wilkinson and Mitchell, 2010). In addition, the relative contribution of organisms to ecosystem functioning does not necessarily fully align with their relative abundance or standing biomass but also depends on their biomass turnover rate (Odum and Barrett, 1971), and this is also the case for soil protists (Wilkinson and Mitchell, 2010). Even if we expect standing biomass and biomass turnover to be correlated (Keeling and Phillips, 2007), it would be interesting for future studies to quantify how the biomass turnover rate of soil protists varies with elevation (Wilkinson and Mitchell, 2010).

The relatively high abundance of parasite reads suggests that they could substantially regulate macro‐organism population dynamics (Poulin, 2011; Mahé *et al*., 2017). If parasites selectively infect specific host lineages, they have in theory the potential to foster local host diversity by limiting population growth of particular host species that are locally abundant. Also, parasites can trigger host diversification and evolve in parallel through a series of Red Queen processes, i.e. the Janzen–Connell hypothesis applied to animals (Mahé *et al*., 2017). The degree of specificity in parasites varies across clades, with Gregarinomorphea usually assumed to be specific (Smith and Cook, 2008; Rueckert *et al*., 2019) while this is not as clear for Oomycota (Jiang and Tyler, 2012; Thines, 2018) and Phytomyxea (Neuhauser *et al*., 2014; Bass *et al*., 2018). While consumers dominated the regional pool of soil protists, we found large local variations: consumers represented between 17% and 89% of the sample read counts. These pronounced local variations in functional composition have also been observed in previous studies. For example, Mahé *et al*. (2017) found substantial variation in the protist parasites relative counts across forest soil samples but did not explore how environmental factors correlated to these variations (e.g. in soil properties). The local variability we observed in this study related to elevation and a number of correlated environmental factors (soil pH, bulk soil water content, plant communities).

Several competing theories predict such variations: one hypothesis posits that parasite counts should decrease with elevation as host total biomass (and diversity) generally decreases above a certain elevation (van Riper *et al*., 1986; Zamora‐Vilchis *et al*., 2012), for instance, plants (Körner, 1999) or insects (Hodkinson, 2005). Another hypothesis posits that parasites counts should increase with elevation as host organisms tend to be more physiologically stressed in a harsher environment and invest relatively more resources to survive in these environmentally stressful high alpine environments and less to defend themselves against parasites (Oppliger *et al*., 1998). Our data validate the first hypothesis: on average, the consumer/parasite reads ratio increases from ca. 3 at low elevation (400–1000 m) to ca. 12 at high elevation (2000–3000 m). We note that due to the compositionality nature of environmental DNA data (Gloor *et al*., 2017; Knight *et al*., 2018), the increase in consumer/parasite ratio toward mountain tops can be either interpreted as (i) a decrease in parasites (consumer remaining constant), (ii) a simultaneous increase in consumers and decrease in parasites, (iii) an increase in consumers (parasite remaining constant), or (iv) a simultaneous decrease in consumers and parasites with a more pronounced decrease for parasites. While interpretations (i), (ii) and (iv) are in line with our first hypothesis of how parasites abundance should relate to elevation, interpretation (iii) is more problematic. Indeed, this would mean an absolute decrease of consumers toward more productive lowland sites, a highly counter‐intuitive result. Hence, we suggest that the *relative* increase of consumers over parasites with elevation is the result of an *absolute* decrease of parasite with elevation.

The growth and reproduction of organisms are not directly influenced by elevation but depends on other ecologically meaningful factors that correlate with elevation, in particular climate and soil properties (Körner, 1999). In our study, high elevation sites tended to be colder and drier, and have calcareous and more alkaline soils. The correlation we found between protist functional composition and elevation could not be unambiguously attributed to one specific environmental factor, like soil pH, bulk soil water content or temperature, as those had confounding effects on functional composition. To disentangle the effects of various climatic and edaphic factors, we suggest that further studies should replicate our sampling scheme along multiple independent elevation gradients and/or design a sampling scheme with a balanced set of alkaline and acidic soil at each elevation and/or make use of experimental designs targeted to answer specific questions within the larger context of climate and edaphic factors. Nonetheless, we found that some plant community composition [approximated here with plant phytosociological alliances, (Yashiro *et al*., 2018)] correlated with protist functional composition, independently of elevation, climatic and edaphic factors measured in this study. For example, sites colonized by plants from the *Petasition paradoxi* alliance (scree) tended to harbour relatively more consumers while sites colonized by plants from the *Calthion* alliance (temporally flooded wet meadows) tended to harbour relatively more parasites. These intriguing correlations might be the product of indirect association between specific plants species and specific soil protists [as is the case between plants and other microbes; e.g. with bacteria in the same area; (Yashiro *et al*., 2018)] but could also reflect the effect of other micro‐edaphic factors that correlated with vegetation type and were not included in our study (Leff *et al*., 2018; Ohlmann *et al*., 2018). For instance, soils of the *Calthion* alliance are water‐logged and anoxic, and thereby selects for plant species and bacterial communities adapted to such conditions (e.g. methanotrophs and anaerobes) (Yashiro *et al*., 2018). Future *in situ* functional studies within the protist parasites group could reveal whether parasitism is what is selected for in *Calthion* sites or whether other functional aptitudes that selectively promote survival in wet anoxic ecosystems are causing increasing relative abundances of the parasites functional group in our study. In addition, it will be key for future studies to document the temporal dynamic of protist functional groups abundance, as it is well established that protist communities exhibit seasonal variation, for example in water (Simon *et al*., 2015) and soil (Fournier *et al*., 2020). Even if soil protist communities seem to vary more spatially than temporally (Fournier *et al*., 2020), we acknowledge that some of the spatial variation reported in this study could reflect temporal dynamics as the sites were sampled at different dates. Future studies could use seasonal variation to better understand parasite abundance dynamics, for example by comparing parasites relative read counts to host biomass and diversity throughout the season.

Finally, we documented how the taxonomic identity of the protists within functional groups responds to the environmental gradient in alpine soils. In other systems, it has been shown that, while functional group composition is often strongly related to environmental gradients, trends in the taxonomic composition within functional groups appear much more difficult to detect, for example bacteria in the global ocean (Louca *et al*., 2016). It has been suggested that these contrasting patterns could result from functional redundancy of organisms within but not between functional groups (Louca *et al*., 2018). In contrast, here we show that the taxonomic composition within each of the three functional groups of protists varied strongly and predictably along the environmental gradient. We found that climatic and edaphic factors explained up to 40% of the within‐group protist taxonomic composition. Interestingly, functional groups presented contrasted responses to the environmental gradients. Theory predicts that host‐associated (e.g. parasite) community composition should be correlated to their host community composition while non‐host‐associated (e.g. phototrophs) taxonomic community composition should be more correlated to the abiotic environments (e.g. soil pH) (Ohlmann *et al*., 2018). Our data do not support this hypothesis: for example, the correlation between plant and protist taxonomic composition was not drastically higher for parasites than for consumers and phototrophs. Interestingly, we found that plant parasite (most of the Oomycota reads) taxonomic composition was correlated to soil pH while animal parasite (Gregarinomorphea) taxonomic composition was more correlated to elevation. The latter result can be interpreted as an indirect effect of animal host turnover along the elevation gradient because (i) Gregarinomorphea are usually considered as relatively host‐specific (Mahé *et al*., 2017; del Campo *et al*., 2019; Singer *et al*., 2019) and (ii) soil animal communities change along elevation gradients (Hodkinson, 2005). The fact that plant parasite communities were correlated to soil pH and TOC can be explained by two non‐mutually exclusive hypotheses. First, individual parasites might be sensitive to abiotic conditions (e.g. soil, climate) during the phase of their life exposed to the outside environment. This is for example the case when they disperse between hosts (i.e. at the zoospore stage) and are not living within host bodies (Thines, 2018). Second, soil pH indirectly affects the presence and/or abundance of their host which could, in turn, affect the distribution of parasite DNA found in soil. For example, plant species distributions and abundances are known to vary with soil pH (Buri *et al*., 2020) so that the distribution of protists that parasitize plants could also be related to soil pH. However, as Oomycota are relatively less host‐specific (Thines, 2018) and the DNA marker used here is not as specific as it is for animal parasites (i.e. it does not allow distinguishing closely related taxa) the second hypothesis seems less likely than the first one. We suggest future ecological studies could improve our understanding of the host–parasite association by better documenting (i) the interaction between the partners, and (ii) the host and (iii) the parasite identity. For example, contrasting the distribution of multiple lineage of parasites represents a fertile ground to better understand the ecology and evolution of protist parasites and their hosts (Poulin, 2011). Retrieving parasite DNA from the soil provides only a little evidence for a direct association with a specific host. We suggest that future studies could directly sample host tissue (e.g. roots) to provide (i) more direct evidence of the interaction as well as (ii) a better idea of the host identity. (iii) Current amplicon approaches based on one unique primer set provide limited information on rare parasites (e.g. the very low abundance of endomyxea and cryptomycota prevented us to analyse them statistically here). We suggest that focusing on multiple parasitic lineages at the same time using different sets of PCR primer targeting different lineages (e.g. as in Bass *et al*., 2018) represents a valuable approach to better document the breath of parasite diversity.

## Author's Contribution

FM developed the idea of this article, run all bioinformatic analysis, interpreted the results with help from EL and wrote the first version of the manuscript. AG and HN‐H conceived and initiated the overall project and field campaign, with help of EY and EP. EY designed the sampling campaign procedure with help from AG and HN‐H. EY, EP and AB collected and processed the soil sample. DS, EL and EADM planned the protist sequencing. DS processed the extracted DNA in preparation for MiSeq sequencing for protist. HM and NG formatted the data with help from EY and LM. AG, EL and EADM co‐financed the protist part of the project with help from HN‐H, EY, EP. AG, HN‐H, EY co‐financed the sampling campaign, soil processing and DNA extraction. All authors provided feedback on the manuscript.

## Availability of Data and Materials

Raw sequences (fastq files) are available on European Nucleotide Archive via the project number PRJEB30010 (ERP112373). The data generated or analysed during this study are included in this published article: the ASV table counts, ASV taxonomy and functions and sample metadata are provided as additional files. The bioinformatic pipeline code is published on *github*: https://github.com/FloMazel/Protist_Altitude_alps.

## Supporting information


**Additional File 1.** Supplementary figure and small tables.

**Additional File 2.** Metadata.

**Additional File 3.** ASV table.

**Additional File 4.** ASV taxonomy and functions.

**Additional File 5.** Rules to infer function from taxonomy.

## References

[emi15686-bib-0001] Adl, M.S. , and Gupta, V.V.S.R. (2006) Protists in soil ecology and forest nutrient cycling. Can J For Res 36: 1805–1817.

[emi15686-bib-0002] Adl, S.M. , Bass, D. , Lane, C.E. , Lukeš, J. , Schoch, C.L. , Smirnov, A. , *et al*. (2019) Revisions to the classification, nomenclature, and diversity of eukaryotes. J Eukaryot Microbiol 66: 4–119. 30257078 10.1111/jeu.12691 PMC6492006

[emi15686-bib-0003] Bardgett, R.D. , and Van Der Putten, W.H. (2014) Belowground biodiversity and ecosystem functioning. Nature 515: 505–511. 25428498 10.1038/nature13855

[emi15686-bib-0004] Bass, D. , van der Gast, C. , Thomson, S. , Neuhauser, S. , Hilton, S. , and Bending, G.D. (2018) Plant rhizosphere selection of plasmodiophorid lineages from bulk soil: the importance of “hidden” diversity. Front Microbiol 9: 168. 29503632 10.3389/fmicb.2018.00168 PMC5825890

[emi15686-bib-0005] Bonkowski, M. (2004) Protozoa and plant growth: the microbial loop in soil revisited. New Phytol 162: 617–631. 33873756 10.1111/j.1469-8137.2004.01066.x

[emi15686-bib-0006] Buri, A. , Grand, S. , Yashiro, E. , Adatte, T. , Spangenberg, J.E. , Pinto‐Figueroa, E. , *et al*. (2020) What are the most crucial soil variables for predicting the distribution of mountain plant species? A comprehensive study in the Swiss Alps. J Biogeogr 47: 1143–1153.

[emi15686-bib-0007] Callahan, B.J. , McMurdie, P.J. , Rosen, M.J. , Han, A.W. , Johnson, A.J.A. , and Holmes, S.P. (2016) DADA2: high‐resolution sample inference from Illumina amplicon data. Nat Methods 13: 581–583. 27214047 10.1038/nmeth.3869 PMC4927377

[emi15686-bib-0008] del Campo, J. , Heger, T.J. , Rodríguez‐Martínez, R. , Worden, A.Z. , Richards, T.A. , Massana, R. , and Keeling, P.J. (2019) Assessing the diversity and distribution of apicomplexans in host and free‐living environments using high‐throughput amplicon data and a phylogenetically informed reference framework. Front Microbiol 10: 2373. 31708883 10.3389/fmicb.2019.02373 PMC6819320

[emi15686-bib-0009] Delarze, R. , Gonseth, Y. , and Galland, P. (1998) Guide des milieux de Suisse. Ecologie – menaces – especes caracteristiques. Lausanne, Switzerland: Delachaux & Niestle.

[emi15686-bib-0010] Dubuis, A. , Pottier, J. , Rion, V. , Pellissier, L. , Theurillat, J.P. , and Guisan, A. (2011) Predicting spatial patterns of plant species richness: a comparison of direct macroecological and species stacking modelling approaches. Divers Distrib 17: 1122–1131.

[emi15686-bib-0011] Fierer, N. (2017) Embracing the unknown: disentangling the complexities of the soil microbiome. Nat Rev Microbiol 15: 579–590. 28824177 10.1038/nrmicro.2017.87

[emi15686-bib-0012] Fiore‐Donno, A.M. , Richter‐Heitmann, T. , and Bonkowski, M. (2020) Contrasting responses of Protistan plant parasites and phagotrophs to ecosystems, land management and soil properties. Front Microbiol 11: 1823. 32849427 10.3389/fmicb.2020.01823 PMC7422690

[emi15686-bib-0013] Fiore‐Donno, A.M. , Richter‐Heitmann, T. , Degrune, F. , Dumack, K. , Regan, K.M. , Marhan, S. , *et al*. (2019) Functional traits and spatio‐temporal structure of a major group of soil protists (Rhizaria: Cercozoa) in a temperate grassland. Front Microbiol 10: 1332. 31244819 10.3389/fmicb.2019.01332 PMC6579879

[emi15686-bib-0014] Foissner, W. (1999) Soil protozoa as bioindicators: pros and cons, methods, diversity, representative examples. In Invertebrate Biodiversity as Bioindicators of Sustainable Landscapes. Amsterdam, The Netherlands: Elsevier, pp. 95–112.

[emi15686-bib-0015] Fournier, B. , Samaritani, E. , Frey, B. , Seppey, C.V.W. , Lara, E. , Heger, T.J. , and Mitchell, E.A.D. (2020) Higher spatial than seasonal variation in floodplain soil eukaryotic microbial communities. Soil Biol Biochem 147: 107842.

[emi15686-bib-0016] Gao, Z. , Karlsson, I. , Geisen, S. , Kowalchuk, G. , and Jousset, A. (2019) Protists: puppet masters of the rhizosphere microbiome. Trends Plant Sci 24: 165–176. 30446306 10.1016/j.tplants.2018.10.011

[emi15686-bib-0017] Geisen, S. (2016) The bacterial‐fungal energy channel concept challenged by enormous functional versatility of soil protists. Soil Biol Biochem 102: 22–25.

[emi15686-bib-0018] Geisen, S. (2018) Soil protists: a fertile frontier in soil biology research. FEMS Microbiol Rev 42: 293–323. 29447350 10.1093/femsre/fuy006

[emi15686-bib-0019] Geisen, S. , Lara, E. , Mitchell, E.A.D. , Völcker, E. , and Krashevska, V. (2020) Soil protist life matters! Soil Org 92: 189–196.

[emi15686-bib-0020] Geisen, S. , Mitchell, E.A.D. , Wilkinson, D.M. , Adl, S. , Bonkowski, M. , Brown, M.W. , *et al*. (2017) Soil protistology rebooted: 30 fundamental questions to start with. Soil Biol Biochem 111: 94–103.

[emi15686-bib-0021] Gloor, G.B. , Macklaim, J.M. , Pawlowsky‐Glahn, V. , and Egozcue, J.J. (2017) Microbiome datasets are compositional: and this is not optional. Front Microbiol 8: 2224. 29187837 10.3389/fmicb.2017.02224 PMC5695134

[emi15686-bib-0022] Glücksman, E. , Bell, T. , Griffiths, R.I. , and Bass, D. (2010) Closely related protist strains have different grazing impacts on natural bacterial communities. Environ Microbiol 12: 3105–3113. 20602629 10.1111/j.1462-2920.2010.02283.x

[emi15686-bib-0023] Guzman, L.M. , Germain, R.M. , Forbes, C. , Straus, S. , O'Connor, M.I. , Gravel, D. , *et al*. (2019) Towards a multi‐trophic extension of metacommunity ecology. Ecol Lett 22: 19–33. 30370702 10.1111/ele.13162

[emi15686-bib-0024] Hadfield, J.D. (2010) MCMC methods for multi‐response generalized linear mixed models: the MCMCglmm R package. J Stat Softw 33: 1–22.20808728

[emi15686-bib-0025] Hijmans, R.J. , Cameron, S.E. , Parra, J.L. , Jones, P.G. , and Jarvis, A. (2005) Very high resolution interpolated climate surfaces for global land areas. Int J Climatol 25: 1965–1978.

[emi15686-bib-0026] Hodkinson, I.D. (2005) Terrestrial insects along elevation gradients: species and community responses to altitude. Biol Rev 80: 489–513. 16094810 10.1017/s1464793105006767

[emi15686-bib-0027] Hoorn, C. , Perrigo, A. , and Antonelli, A. (2018) Mountains, Climate and Biodiversity. Hoboken, NJ: John Wiley & Sons.

[emi15686-bib-0028] Jiang, R.H.Y. , and Tyler, B.M. (2012) Mechanisms and evolution of virulence in oomycetes. Annu Rev Phytopathol 50: 295–318. 22920560 10.1146/annurev-phyto-081211-172912

[emi15686-bib-0029] Keeling, H.C. , and Phillips, O.L. (2007) The global relationship between forest productivity and biomass. Glob Ecol Biogeogr 16: 618–631.

[emi15686-bib-0030] Knight, R. , Vrbanac, A. , Taylor, B.C. , Aksenov, A. , Callewaert, C. , Debelius, J. , *et al*. (2018) Best practices for analysing microbiomes. Nat Rev Microbiol 16: 410–422. 29795328 10.1038/s41579-018-0029-9

[emi15686-bib-0031] Koller, R. , Rodriguez, A. , Robin, C. , Scheu, S. , and Bonkowski, M. (2013) Protozoa enhance foraging efficiency of arbuscular mycorrhizal fungi for mineral nitrogen from organic matter in soil to the benefit of host plants. New Phytol 199: 203–211. 23534902 10.1111/nph.12249

[emi15686-bib-0032] Körner, C. (1999) Alpine Plant Life: Functional Plant Ecology of High Mountain Ecosystems. Berlin: Springer.

[emi15686-bib-0033] Large, E.C. (1940) The Advance of the Fungi. London: Jonathan Cape.

[emi15686-bib-0034] Leff, J.W. , Bardgett, R.D. , Wilkinson, A. , Jackson, B.G. , Pritchard, W.J. , De Long, J.R. , *et al*. (2018) Predicting the structure of soil communities from plant community taxonomy, phylogeny, and traits. Nature 12: 1794–1805. 10.1038/s41396-018-0089-x PMC6004312 29523892

[emi15686-bib-0035] Louca, S. , Parfrey, L.W. , and Doebeli, M. (2016) Decoupling function and taxonomy in the global ocean microbiome. Science 353: 1272–1277. 27634532 10.1126/science.aaf4507

[emi15686-bib-0036] Louca, S. , Polz, M.F.M. , Mazel, F. , Albright, M.B.N.M. , Julie, H. , O'Connor, M. , *et al*. (2018) Function and functional redundancy in microbial systems. Nat Ecol Evol 2: 936–943. 29662222 10.1038/s41559-018-0519-1

[emi15686-bib-0037] Mahé, F. , De Vargas, C. , Bass, D. , Czech, L. , Stamatakis, A. , Lara, E. , *et al*. (2017) Parasites dominate hyperdiverse soil protist communities in neotropical rainforests. Nat Ecol Evol 1: 91. 28812652 10.1038/s41559-017-0091

[emi15686-bib-0038] Margerison, R. , Nicolitch, O. , and Zhang, Y. (2020) Microbiomes of soils. In Microbiomes of Soils, Plants and Animals, Antwis, R.E. , Harrison, X.A. , and Cox, M.J. (eds). Cambridge, MA: Cambridge University Press, pp. 29–54.

[emi15686-bib-0039] Martin, M. (2011) Cutadapt removes adapter sequences from high‐throughput sequencing reads. EMBnet J 17: 10.

[emi15686-bib-0040] McMurdie, P.J. , and Holmes, S. (2013) Phyloseq: an R package for reproducible interactive analysis and graphics of microbiome census data. PLoS One 8: e61217. 23630581 10.1371/journal.pone.0061217 PMC3632530

[emi15686-bib-0041] Neuhauser, S. , Kirchmair, M. , Bulman, S. , and Bass, D. (2014) Cross‐kingdom host shifts of phytomyxid parasites. BMC Evol Biol 14: 1–13. 24559266 10.1186/1471-2148-14-33 PMC4016497

[emi15686-bib-0042] Odum, E.P. , and Barrett, G.W. (1971) Fundamentals of Ecology. Philadelphia: Saunders.

[emi15686-bib-0043] Ohlmann, M. , Mazel, F. , Chalmandrier, L. , Bec, S. , Coissac, E. , Gielly, L. , *et al*. (2018) Mapping the imprint of biotic interactions on β‐diversity. Ecol Lett 21: 1660–1669. 30152092 10.1111/ele.13143

[emi15686-bib-0044] Oksanen, J. , Blanchet, F.G. , Roeland, K. , Legendre, P. , Minchin, P.R. , O'Hara, R.B. , et al. (2016) Vegan: Community Ecology Package. R package version 2.3‐4. http://CRAN.R-project.org/package=vegan.

[emi15686-bib-0045] Oliverio, A.M. , Geisen, S. , Delgado‐Baquerizo, M. , Maestre, F.T. , Turner, B.L. , and Fierer, N. (2020) The global‐scale distributions of soil protists and their contributions to belowground systems. Sci Adv 6: eaax8787. 32042898 10.1126/sciadv.aax8787 PMC6981079

[emi15686-bib-0046] Oppliger, A. , Clobert, J. , Lecomte, J. , Lorenzon, P. , Boudjemadi, K. , and John‐Alder, H.B. (1998) Environmental stress increases the prevalence and intensity of blood parasite infection in the common lizard *Lacerta vivipara* . Ecol Lett 1: 129–138.

[emi15686-bib-0047] Poulin, R. (2011) Evolutionary Ecology of Parasites. Princeton, NJ: Princeton University Press.

[emi15686-bib-0048] R Development Core Team . (2015) R: A Language and Environment for Statistical Computing. Vienna: Austria.

[emi15686-bib-0049] Rahbek, C. , Borregaard, M.K. , Colwell, R.K. , Dalsgaard, B. , Holt, B.G. , Morueta‐Holme, N. , *et al*. (2019) Humboldt's enigma: what causes global patterns of mountain biodiversity? Science 365: 1108–1113. 31515383 10.1126/science.aax0149

[emi15686-bib-0050] Rueckert, S. , Betts, E.L. , and Tsaousis, A.D. (2019) The symbiotic spectrum: where do the gregarines fit? Trends Parasitol 35: 687–694. 31345767 10.1016/j.pt.2019.06.013

[emi15686-bib-0051] Sanders, D. , Thébault, E. , Kehoe, R. , and Frank van Veen, F.J. (2018) Trophic redundancy reduces vulnerability to extinction cascades. Proc Natl Acad Sci U S A 115: 2419–2424. 29467292 10.1073/pnas.1716825115 PMC5878001

[emi15686-bib-0052] Schulz, G. , Schneider, D. , Brinkmann, N. , Edy, N. , Daniel, R. , Polle, A. , *et al*. (2019) Changes in trophic groups of protists with conversion of rainforest into rubber and oil palm plantations. Front Microbiol 10: 240. 30809219 10.3389/fmicb.2019.00240 PMC6380168

[emi15686-bib-0053] Seppey, C.V.W. , Broennimann, O. , Buri, A. , Yashiro, E. , Pinto‐Figueroa, E. , Singer, D. , *et al*. (2020) Soil protist diversity in the Swiss western Alps is better predicted by topo‐climatic than by edaphic variables. J Biogeogr 47: 866–878.

[emi15686-bib-0054] Seppey, C.V.W. , Singer, D. , Dumack, K. , Fournier, B. , Belbahri, L. , Mitchell, E.A.D. , and Lara, E. (2017) Distribution patterns of soil microbial eukaryotes suggests widespread algivory by phagotrophic protists as an alternative pathway for nutrient cycling. Soil Biol Biochem 112: 68–76.

[emi15686-bib-0055] Simon, M. , López‐García, P. , Deschamps, P. , Moreira, D. , Restoux, G. , Bertolino, P. , and Jardillier, L. (2015) Marked seasonality and high spatial variability of protist communities in shallow freshwater systems. ISME J 9: 1941–1953. 25853803 10.1038/ismej.2015.6 PMC4542043

[emi15686-bib-0056] Singer, D. , Duckert, C. , Heděnec, P. , Lara, E. , Hiltbrunner, E. , and Mitchell, E.A.D. (2020) High‐throughput sequencing of litter and moss eDNA reveals a positive correlation between the diversity of Apicomplexa and their invertebrate hosts across alpine habitats. Soil Biol Biochem 147: 107837.

[emi15686-bib-0057] Singer, D. , Metz, S. , Unrein, F. , Shimano, S. , Mazei, Y. , Mitchell, E.A.D. , and Lara, E. (2019) Contrasted micro‐eukaryotic diversity associated with *Sphagnum* mosses in tropical, subtropical and temperate climatic zones. Microb Ecol 78: 714–724. 30756135 10.1007/s00248-019-01325-7

[emi15686-bib-0058] Smith, A.J. , and Cook, T.J. (2008) Host specificity of five species of Eugregarinida among six species of cockroaches (Insecta: Blattodea). Comp Parasitol 75: 288–291.

[emi15686-bib-0059] Stoeck, T. , Bass, D. , Nebel, M. , Christen, R. , Jones, M.D.M. , Breiner, H.W. , and Richards, T.A. (2010) Multiple marker parallel tag environmental DNA sequencing reveals a highly complex eukaryotic community in marine anoxic water. Mol Ecol 19: 21–31. 20331767 10.1111/j.1365-294X.2009.04480.x

[emi15686-bib-0060] Taberlet, P. , Bonin, A. , Zinger, L. , and Coissac, E. (2018) Environmental DNA: For Biodiversity Research and Monitoring. Oxford, UK: Oxford University Press.

[emi15686-bib-0061] Thines, M. (2018) Oomycetes. Curr Biol 28: R812–R813. 30086308 10.1016/j.cub.2018.05.062

[emi15686-bib-0062] van Riper, C. , van Riper, S.G. , Goff, M.L. , and Laird, M. (1986) The epizootiology and ecological significance of malaria in Hawaiian land birds. Ecol Monogr 56: 327–344.

[emi15686-bib-0063] von Humbolt, A. , and Bonpland, A. (1805) Essai sur la géographie des plantes: accompagné d'un tableau physique des régions équinoxiales, fondé sur des mesures exécutées, depuis le dixième degré de latitude boréale jusqu'au dixième degré de latitude australe, pendant les années 1799, 1800, 1801. Paris: Levrault Schoell.

[emi15686-bib-0064] Wickham, H. , Averick, M. , Bryan, J. , Chang, W. , McGowan, L. , François, R. , *et al*. (2019) Welcome to the Tidyverse. J Open Source Softw 4: 1686.

[emi15686-bib-0065] Wilkinson, D.M. , and Mitchell, E.A.D. (2010) Testate amoebae and nutrient cycling with particular reference to soils. Geomicrobiol J 27: 520–533.

[emi15686-bib-0066] Xiong, W. , Jousset, A. , Guo, S. , Karlsson, I. , Zhao, Q. , Wu, H. , *et al*. (2018) Soil protist communities form a dynamic hub in the soil microbiome. ISME J 12: 634–638. 29028001 10.1038/ismej.2017.171 PMC5776453

[emi15686-bib-0067] Yashiro, E. , Pinto‐Figueroa, E. , Buri, A. , Spangenberg, J.E. , Adatte, T. , Niculita‐Hirzel, H. , *et al*. (2016) Local environmental factors drive divergent grassland soil bacterial communities in the western Swiss Alps. Appl Environ Microbiol 82: 6303–6316. 27542929 10.1128/AEM.01170-16 PMC5066347

[emi15686-bib-0068] Yashiro, E. , Pinto‐Figueroa, E. , Buri, A. , Spangenberg, J.E. , Adatte, T. , Niculita‐Hirzel, H. , *et al*. (2018) Meta‐scale mountain grassland observatories uncover commonalities as well as specific interactions among plant and non‐rhizosphere soil bacterial communities. Sci Rep 8: 5758. 29636506 10.1038/s41598-018-24253-x PMC5893626

[emi15686-bib-0069] Zamora‐Vilchis, I. , Williams, S.E. , and Johnson, C.N. (2012) Environmental temperature affects prevalence of blood parasites of birds on an elevation gradient: implications for disease in a warming climate. PLoS One 7: e39208. 22723966 10.1371/journal.pone.0039208 PMC3378574

