# Supplementary material for: Soil protist function varies with elevation in the Swiss Alps
Source: Environ Microbiol. 2021 Aug 10;24(4):1689–702. doi: 10.1111/1462-2920.15686 (PMC9290697; doi:10.1111/1462-2920.15686)
Supplement: Supplementary file 1 — Additional File 1. Supplementary figure and small tables. [file EMI-24-1689-s005.docx]

**Supplementary Table 2.** Functional group relative abundance ratio is correlated to elevation and environment. The table reports the result of the comparison between a full model and a reduced model for each environmental factor. The full model uses all the environmental factors as explanatory variables and the ratio of consumer to parasite reads as the variable to explain (full model). A reduced model was built for each environmental factor by dropping the given factor. The comparison between the fit of the full model and each of the reduced models was done using a F-test and is given in the table (F-values and associated p-values along with Total sum of squares, Residual sum of squares (RSS) and AIC).

**Supplementary Figure 1.** **Protist lineage relative readcounts relates to the environment.** All panels depict the relationship between the relative abundance (relative number of reads per sample) of some lineages of parasites (panel A) and consumers (panel B) and the elevation (meters ASL). Points are colored by plant richness.

**Supplementary Figure 2.** **Environmental variability of the study region.** The figure presents the two first axis of a principal component analysis that included seven edaphic variable and 26 climatic variables (BIO1 = Annual Mean Temperature, BIO2 = Mean Diurnal Range (Mean of monthly (max temp - min temp)), BIO3 = Isothermality (BIO2/BIO7) (×100), BIO4 = Temperature Seasonality (standard deviation ×100), BIO5 = Max Temperature of Warmest Month, BIO6 = Min Temperature of Coldest Month, BIO7 = Temperature Annual Range (BIO5-BIO6), BIO8 = Mean Temperature of Wettest Quarter, BIO9 = Mean Temperature of Driest Quarter, BIO10 = Mean Temperature of Warmest Quarter, BIO11 = Mean Temperature of Coldest Quarter, BIO12 = Annual Precipitation, BIO13 = Precipitation of Wettest Month, BIO14 = Precipitation of Driest Month, BIO15 = Precipitation Seasonality (Coefficient of Variation), BIO16 = Precipitation of Wettest Quarter, BIO17 = Precipitation of Driest Quarter, BIO18 = Precipitation of Warmest Quarter, BIO19 = Precipitation of Coldest Quarter).

**Supplementary Figure 3.** **Correlations between the selected environmental factors.** The figure shows the pairwise correlations between the selected environmental factors. The upper triangle presents each pairwise spearman correlation.

**Supplementary Figure 4.** **Consumer and parasite abundance vary widely with elevation.** Panel A presents the variability of the Consumer/Parasites reads counts (Y-axis, on a log scale) along the altitudinal gradient (X-axis). Points are colored by plant alliance and a smooth fit was added. Panel B present boxplots of the residuals from the smooth fit depicted in panel A (Y-axis) across the different plant alliances. Points are colored according to water content (on a log scale).

**Supplementary Figure 5.** **Community composition within functional groups across the altitudinal gradient.** The figure shows how taxonomic (ASV) compositional turnover (beta-diversity measure = Bray Curtis) varies across the environmental gradient within each of the three functional groups (consumers, parasites, phototrophs). Panel A-C depict NMDS ordination of beta diversity with samples colored by elevation for the three functional groups independently. Panel D-E depict the results of distance-based redundancy analysis based on standardized Bray Curtis dissimilarities. Panel E presents the general fit of the model (R^2^) and panel D presents individual factor importance, as assessed by marginal pseudo F-values (i.e. effect of the factor considering all other factors in the model). Statistical significance of each factor is calculated using 999 permutations and indicated with an asterisk (*: p-value <.05, ** p-value <.01). The figure is similar to the Figure 2 in the main text but has been produced with a rarefied ASV table (350 reads).

**Supplementary Figure 6.** **Community composition within parasites across the altitudinal gradient.** The figure is similar to the Figure 6 in the main text but has been produced with a rarefied ASV table (350 reads). The figure shows how taxonomic (ASV) compositional turnover (beta-diversity measure = Bray Curtis) varies across the environmental gradient within each of two parasitic clades. Panel A-B depict NMDS ordination of beta diversity with samples colored by elevation for the two clades independently. Panel C-D depict the results of distance-based redundancy analysis based on Bray Curtis dissimilarities. Panel D presents the general fit of the model (R^2^) and panel C presents individual factor importance, as assessed by marginal pseudo F-values (i.e. effect of the factor considering all other factors in the model). Statistical significance of each factor is calculated using 999 permutations and indicated with an asterisk (*: p-value <.05, ** p-value <.01).

**Supplementary Figure 7.** **Genus-level community composition of parasites.** Panel A presents the relative read counts of Gregarinomorphea genera across three altitudinal bins. Panel B presents the relative read counts of Oomycota genera across pH bins. Sample reads counts are first normalized by sample depth, then all samples from a given bin are pooled together and normalized by the number of samples in that bin.

**Supplementary Figure 8.** **Read counts and distribution of Phytomyxea (Endomyxa).** Panel A presents the distribution of read counts per sample for Phytomyxea across all samples. Panel B presents the relative read counts of Phytomyxea genera across altitude bins. Sample reads counts are first normalized by sample depth, then all samples from a given bin are pooled together and normalized by the number of samples in that bin.
